# Personalised cancer follow-up: risk stratification, needs assessment or both?

**DOI:** 10.1038/bjc.2011.535

**Published:** 2012-01-03

**Authors:** E K Watson, P W Rose, R D Neal, N Hulbert-Williams, P Donnelly, G Hubbard, J Elliott, C Campbell, D Weller, C Wilkinson

**Affiliations:** 1 Faculty of Health and Life Sciences, Oxford Brookes University, Oxford OX3 OFL, UK; 2 Department of Primary Health Care, University of Oxford, Oxford OX1 2ET, UK; 3 North Wales Centre for Primary Care Research, North Wales Clinical School, College of Health & Behavioural Sciences, Bangor University, Wrexham LL13 7YP, UK; 4 Department of Psychology, University of Chester, Chester, CH1 4BJ, UK; 5 Breast Care Directorate, South Devon Healthcare Foundation Trust, Torbay Hospital, Torbay, UK; 6 Cancer Care Research Centre, School of Nursing, Midwifery and Health, University of Stirling, Highland Campus, Inverness, UK; 7 Consultant Research Analyst, National Cancer Survivorship Initiative Consequences of Treatment Programme, Macmillan Cancer Support, 89 Albert Embankment, London SE1 7UQ, UK; 8 School of Clinical Sciences and Community Health, Centre for Population Health Sciences, University of Edinburgh, Edinburgh EH8 9AG, UK

There are approximately 2 million people now living with or beyond cancer in the UK (Maddams *et al*, 2009) and this number is increasing. Cancer survivors can experience physical, psychological and social consequences as a result of the disease and the treatments received (Jefford *et al*, 2008; Foster *et al*, 2009). The effects may be immediate, some of which will resolve and others may persist and become long-term. Late effects can also occur and the interval between the end of treatment and onset can range from a few weeks (e.g. lymphoedema after axillary node removal) to several years (e.g. heart disease following radiotherapy to the chest area). Problems will be individual to each patient due to a unique combination of circumstances including the site and stage of the cancer, the type of treatment(s) given, the age of the patient, genetic factors, concomitant co-morbidities, family and social circumstances, and personality traits.

The recent National Cancer Survivorship Initiative (NCSI) acknowledges the range of issues which cancer survivors may face, and highlights the need for health professionals to organise care accordingly (NCSI, 2010a). The recent NCSI Research Priorities Report (NCSI, 2010b) identified two areas of priority for research to inform practice: the establishment of large cohort studies (to determine the range and frequency of problems following treatment) and the development of risk stratification tools. However, the report did not develop a detailed definition of risk stratification nor did it elucidate what outcomes risk stratification should address.

The purpose of this paper, prepared by members of the Survivorship Sub-group of the National Cancer Research Institute Primary Care Clinical Studies Group, is therefore to further define the term risk stratification in relation to cancer survivorship; to propose a framework for risk stratification; and to consider what research is required to support its implementation. The focus of our paper is on stratifying risk in relation to the late effects of diagnosis and treatment. We do not therefore address prognostic risk stratification, a topic for which there is already a large body of literature.

## Definition of risk

We have defined risk stratification as the process of quantifying the probability of a harmful effect to individuals resulting from a range of internal and external factors (e.g., demographic characteristics, genetic make-up, medical treatments). Risk must be differentiated from (healthcare) need, which is the capacity to benefit from health care. A need must be present at the time of assessment, unlike a risk, which implies something that might happen in the future. The assessment of both risk and need are required in the context of cancer survivorship. The categorisation of outcomes presented in Box 1, which we believe may warrant risk stratification following diagnosis and treatment of cancer, could also apply to needs assessment.

## Risk stratification in cancer survivors

There are already many examples in medicine of clinical prediction tools to assess risk and determine clinical management. For example, Framingham and Q risk scores for cardiovascular risk and the Wells score for deep vein thrombosis. Some also exist in psychology, for example, the Social Readjustment Rating Scale (Holmes and Rahe, 1967) gives a risk score for later onset of stress-related illness on the basis of how many stressor events have been experienced.

The challenge in relation to cancer survivorship is to develop tools for stratifying individual risk across a range of outcomes (medical and psychosocial). This includes assessment of:

- which patients are at risk of which adverse outcomes and the magnitude of the risk;
- the significance of the risk in terms of its’ impact on the individual;
- the availability of effective interventions;
- the health economic implications of managing the risk.

This information will be useful to the health care system to specify the follow-up pathway, and to the patient to enable informed decisions about interventions and life style changes to reduce risk.

Currently, risk stratification in relation to cancer tends to focus on risk of recurrence and is used to inform follow-up pathways. However, there are as yet very few cancers, where there is good evidence to suggest early identification of recurrence reduces mortality: there is evidence for more intensive follow up of colorectal cancer reducing mortality (Renehan *et al*, 2002), but not breast cancer (Rojas *et al*, 2005). There also tends to be insufficient attention to psychosocial dimensions, which can be very important to patients and their families.

We propose that risk stratification should be viewed as a screening test whereby a population at potential risk (all cancer survivors) are offered a test (risk stratification tool) to determine those who need further investigation, intervention or support. Therefore, a modification of the Wilson–Junger criteria (Wilson and Junger, 1968) for an effective screening test could determine which cancer outcomes warrant risk stratification (Box 2). As well as the development of pre-symptomatic interventions, this approach would require additional knowledge of the timing and magnitude of these outcomes in a general population of cancer survivors (as opposed to the population followed up in clinical trials). This may be particularly problematic for psychosocial co-morbidities where the prediction of long-term problems is complex due to the subjective nature of symptoms and the multifaceted nature of variables, which might affect their onset.

Tables 1a and 1b describe the incidence of various treatment effects for breast and prostate cancer, respectively. Although there is some evidence available regarding the incidence of long-term and late effects, this is not always well described and, importantly, there are few proven interventions for those at high risk.

In terms of assessment after treatment is complete, long-term and late effects fall into three categories:

- Those already present and where management will be determined by needs assessment.
- Those problems that are not necessarily present immediately after treatment, but where our criteria for risk assessment are not fulfilled.
- Those that fulfil the modified Wilson and Junger criteria and will therefore qualify for risk stratification.

The first category would include all long-term effects of treatment, for example, urinary and sexual problems resulting from the treatment of prostate cancer. Depression and anxiety may occur at any time and it might be possible to stratify the risk and timing of their occurrence. However, validated screening tools are available, and we suggest a needs assessment approach is most appropriate in this case. The second and third categories are more problematic. For example, the relationship between breast cancer treatment and heart failure is well established (Ewertz and Jensen, 2011) and it is possible to identify those at greatest risk using echocardiography. However, incidence is low and there is no evidence yet that pre-symptomatic treatment affects outcome (Wang *et al*, 2003) so this condition does not fulfil our criteria for risk assessment at present. In the case of osteoporosis following hormone therapy for treatment of prostate cancer, there is a good screening test available (bone mineral density screening) and there is an intervention available to reduce the risk of fractures (bisphosphonates), which is an important health problem (Greenspan *et al*, 2007). A risk assessment and stratification approach could therefore be beneficial in this case. However, there are currently very few late effects that qualify for risk assessment and stratification using these criteria, and we suggest that for now most long-term and late effects will be managed by needs assessment, continuing current practice of managing the problem as it arises. There is a range of screening tools already available for assessing physical and psychological needs, for example, Distress Thermometer, Health Anxiety and Depression Score (HADS) and International Prostate Symptom Score (IPSS).

## Patient perspective

One important issue to consider is the extent to which patients wish to know their risks, especially when there may be few interventions to minimise these risks before they become an overt problem. Risk information can sometimes be complex, and we need to establish the best ways to convey information in a way that patients can understand and retain (Calman and Royston, 1997). There is the potential to cause more harm than benefit. For some, knowledge of future risk may be a significant risk factor for later anxiety, depression and fear. Some patients will prefer to put their cancer experience behind them at the end of treatment, and move on with their lives, and it is important they be allowed to do this (Khan *et al*, 2011). Others may wish to know the risks of recurrence and/or late effects and to take whatever preventive action or to seek help promptly if any problems occur. Currently it is not known when the optimal time is or what the optimal approach is to discuss risks with a patient, and who is best placed to do this.

## A framework for risk stratification and needs assessment following cancer treatment

Risks will vary throughout the cancer trajectory and one single assessment may not be sufficient.

**Figure 1:**
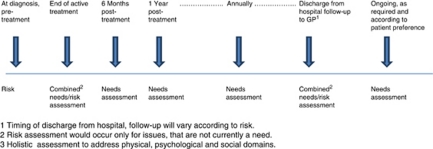
A framework for holistic assessment^3^ of risks and needs.

We propose the first time point for risk stratification is following diagnosis and pre-treatment. At this stage it is possible to stratify to a degree according to the known side-effect profile of certain treatments. Psychosocial factors could also influence the success or compliance with treatment recommendations. The second time point would be on completion of primary treatment. Whereas the ideal would be to have a single tool, in reality it is likely there will be a core tool addressing risks relevant to most cancers, with site-specific additions required. This would inform the follow-up strategy and care plan for the patient, and could be shared with the patient as appropriate. The NCSI is currently proposing that those patients at low risk of recurrence and late effects (physical and psychosocial) would be encouraged towards supported self-management, those at medium risk would have planned, co-ordinated care and those at high risk would receive complex care from specialist services. Needs assessment remains very important during follow-up and should complement risk stratification efforts. For non-complex cases, current cancer follow up usually involves 3–5 years of follow up under the care of a hospital consultant, and we would propose needs (medical and psychosocial) be monitored and addressed at each follow-up appointment. Guidelines should be drawn up to ensure all potential needs are assessed and there is uniformity among different hospitals. Patients are subsequently discharged from hospital follow-up to general practitioner care, and we propose this as the third time point when risk assessment and stratification would be appropriate. The GP (and patient if appropriate) would then be informed regarding possible risks, and the appropriate monitoring, screening and health promotion activities to undertake in the future. Currently patients have a cancer care review with their GP within 6 months of diagnosis. Although this often takes place before the end of active treatment, it does provide an important opportunity for an initial needs assessment in primary care. Additional cancer care reviews could become the vehicle for continuing needs assessment at regular (perhaps annual) intervals in the primary care setting, according to patient preferences (see Figure 1).

## What are the priorities for research?

Research is needed to establish how common late effects are for different cancer sites and treatment regimes, which factors (both physical and psychosocial) are amenable to risk assessment and stratification, how we elicit and assess these risks, what interventions are effective and, importantly, the level of benefit afforded to patients as a result. We also need to develop a better understanding of patients’ preferences in this area and to develop clear and effective strategies for communicating the various risks following treatment to patients and their families. There needs to be robust evaluation of risk stratification tools that are developed, including developing an understanding of factors, which influence compliance with recommended treatments or lifestyle changes.

## Conclusions

Many individuals with cancer can now expect to be cured or to survive for long periods of time. However, survivors can experience a range of short and long-term physical and psychosocial sequelae following treatment, and methods of maximising recovery are needed. Long-term follow-up needs to be rationalised, and to be consistent across centres. Risk stratification, identifying those most likely to have significant problems in the future and intervening accordingly, is an attractive notion, which could inform commissioning of cancer services. The challenge is to individually stratify risk for each potential outcome in order to determine which patients are at risk of which outcomes, and the magnitude of the risk (both in its effect on the individual and the health economic implications). Risk stratification should, however, only be undertaken for outcomes where there are effective interventions, and a holistic approach to needs assessment remains important. For some, perhaps many, outcomes regular needs assessment is currently a more suitable approach than risk stratification.

Long-term follow up involving both needs and risk assessment, addressing both medical and psychosocial needs, is an enormous task and must be underpinned by good research evidence. We suggest that funding agencies plan a research programme immediately that will identify those late effects of cancer and its treatment that are important and amenable to modification to inform future follow-up regimes.

## Figures and Tables

**Table 1a:** Long-term and late effects following breast cancer treatment (Irwig and Bennetts, 1997; Petrek *et al*, 2001; Murphy *et al*, 2007; Ewertz and Jensen, 2011; NHS Information Centre, 2011)

**Long term/late effect**	**Incidence**	**Timing**	**Risk factors**
Shoulder/arm pain	47% 1–3 years (13% severe) 20–30% after 3 years	Immediate	Young age, axillary dissection, radiotherapy
			
Hot flushes	50–70%	Immediate	Young age hormone blocking treatment
			
Lymphoedema	15–25% 1–5 years 1% per year 3–20 years	Immediate and resolving, but some new cases present late	Mastectomy, radiotherapy, axillary dissection, young age
			
Arthralgia	35%	Immediate to 5 years	Tamoxifen Aromatase inhibitors
			
Premature menopause	55% <40 years 90% >40 years		Chemotherapy
			
Osteoporosis	20%	2–5 years	Aromatase inhibitors
			
Cardiovascular disease	0.5–4% following chemotherapy 3% increase in death after radiotherapy per Gy	Death rate increases from 10 years	Chemotherapy, radiotherapy
			
Wound pain or numbness	10%	2 months–5 years	Mastectomy, axilliary gland clearance
			
Poor cosmesis/reconstruction	20% will have reconstruction		
			
Depression	14 *vs* 5% In general population		Young age, previous psychiatric disease, low socio-economic status
			
Sleep disturbance	39%		
			
Fatigue	20–30% at 2 years 5–34% ongoing	Immediate and long term	

**Table 1b:** Long-term and late effects following prostate cancer treatment (Shahinian *et al*, 2005; Ganz, 2009; Smith *et al*, 2009; Harrison *et al*, 2011)

**Treatment effect**	**Incidence**	**Timing**	**Risk factors**
Urinary incontinence	12.3% Following radical prostatectomy at 3 years 2.7% following radiotherapy at 3 years 7% following brachytherapy at 3 years 4.3% following hormone treatment at 3 years	Immediate – can be long term with problems resolving in some instances	Problems more common following radical prostatectomy
			
Bowel problems	3.5% Following radical prostatectomy at 3 years 14.5% following radiation therapy at 3 years 9.3% following brachytherapy at 3 years 6.4% following hormone treatment at 3 years	Sometimes immediate, sometimes delayed, can be long term with problems resolving over time in some instances	Problems more common following radiotherapy
			
Erectile dysfunction	77.4% following radical prostatectomy at 3 years 67.9% following radiotherapy at 3 years 72.1% following brachytherapy at 3 years 97.8% following hormone therapy at 3years	Immediate, often long term. Treatments can sometimes be effective and sometimes the problem will resolve over time	Radical prostatectomy, radiotherapy, hormone treatment
			
Risk of fracture	5 years post-diagnosis, 19.4% of men receiving hormone therapy with fractures compared with 12.6% of those who had not received hormone therapy.	Delayed effect	Hormonal treatment
			
Anxiety/depression		Can occur at any time	Trait anxiety
Hot flushes	Up to 75% of patients		Hormonal treatment

## Box 1 Outcomes following diagnosis and treatment of cancer, which may warrant risk assessment

- *Physical health*
  Recurrence
  Long-term effects of treatment
  Late effects of treatment
  Effect on co-morbidities
  Death
- *Psychological health*
  Increased depression
  Increased anxiety, including fear of recurrence
  Psychosexual problems
  Quality of life
- *Social issues*
  Financial
  Employment
  Education
  Interpersonal
  Social interaction
  Disability

## Box 2 Modification of the Wilson and Junger (1968) criteria for use in risk stratification in cancer survivors

1. The condition/problem should be an important health problem.
2. There should be an effective intervention (e.g., prevention, treatment or supportive mechanism) for the condition/problem (as determined by a randomised controlled trial or as high a level of evidence as possible).
3. Facilities for diagnosis and treatment should be available.
4. There should be a latent stage between diagnosis/treatment and onset.
5. There should be a test, psychometric measure or examination for detection of the condition/problem.
6. The test, measure or examination should be acceptable to the population.
7. The natural history of the condition/problem should be adequately understood.
8. There should be an agreed policy on whom to treat.
9. The total cost of finding a case should be economically balanced in relation to expenditure as a whole.
10. Assessment should be a continuous process, not just a ‘once and for all’ project.
